# Migrate your mind: the role of palliative care in transcultural cancer treatment: A qualitative analysis

**DOI:** 10.1007/s00508-019-1474-9

**Published:** 2019-04-17

**Authors:** Sophie Roider-Schur, Tamara Rumpold, Kathrin Kirchheiner, Eva Katharina Masel, Romina Nemecek, Michaela Amering, Herbert Watzke, Beate Schrank

**Affiliations:** 1 Clinical Division of Palliative Care, Department of Internal Medicine I, Medical University of Vienna, Waehringer Guertel 18–20, 1090 Vienna, Austria; 2 Clinical Division of Oncology, Department of Internal Medicine I, Sankt Josef Hospital Vienna, Auhofstraße 189, 1130 Vienna, Austria; 3 Department of Radiation Oncology, Medical University of Vienna, Vienna, Austria; 4 Department of Dermatology and Tropical Medicine, Military Medical Cluster East, Austrian Armed Forces, Vienna, Austria; 5 Division of Social Psychiatry, Department of Psychiatry and Psychotherapy, Medical University of Vienna, Vienna, Austria; 6 Department of Psychiatry and Psychotherapy, University Hospital Tulln, Karl Landsteiner University of Health Sciences, Tulln, Austria

**Keywords:** Oncology, End of life care, Migrants, Communication, Culture-sensitive care

## Abstract

**Background:**

In increasingly multi-ethnic societies fostering cultural awareness and integration of immigrants is not only a political duty but also an obligation for social and healthcare systems. Importantly, cultural beliefs and needs strongly impact on the quality of life of cancer patients and may become even more crucial at the end of life. However, to date, ethnic and cultural aspects of palliative care are insufficiently researched.

**Methods:**

This qualitative study at the Medical University of Vienna included 21 staff members from different disciplines in oncology and palliative care working with patients with various cultural backgrounds at the end of life. Semi-structured interviews were performed to gain insights into specific aspects of palliative care that are important in the clinical encounter with terminally ill cancer patients with migrant backgrounds and their relatives.

**Results:**

Interviews revealed specific aspects of palliative care, which fell into four fundamental categories and were all perceived as beneficial in the clinical encounter with migrant clients: (A) structural and (B) personal conditions of the palliative care setting, (C) specific care and treatment intentions and (D) personnel requirements and attitudes.

**Conclusion:**

This study revealed first insights into possibilities and prospects of transcultural palliative care for migrants and their relatives. The results might have important implications for the end of life care in this growing population.

## Background

Throughout human history, individuals, families and groups have emigrated from their native homes for many reasons: economic, social or educational advantages, the need to escape war or conflicts, as well as family reunification [1, 2]. Globalization has brought a steady increase in migration, and currently 244 million people worldwide live outside their country of origin [3]. It is well known that migrants who suffer from cancer have worse quality of life (QOL) and psychological outcomes when compared to native populations [2, 4, 5]. Cultural factors are important in relation to the perception of a disease, symptom experience and distress as well as communication and may become even more crucial at the end of life (EOL) [2, 6–8].

The World Health Organization (WHO) defines QOL as “an individual’s perception of their position in life in the context of the culture and value systems in which they live and in relation to their goals, expectations, standards and concerns” [9, 10]. Palliative care (PC) aims to maximize the QOL within the remaining time in an individual’s life [10]. Since, according to the WHO definition, QOL is both subjectively and culturally bound, awareness of transcultural needs of patients and their relatives becomes essential to provide ideal EOL care [7, 8]; however, although experiences in the work with cancer patients with migrant backgrounds have gained social attention and scientific recognition in recent years [4, 6, 11, 12], ethnic and cultural aspects of PC are still underrepresented in the current literature [6].

Using a qualitative research design, this study aimed to gain insights into practical aspects of PC in the clinical encounter with terminally ill cancer patients with migrant backgrounds and their relatives. In recent years, qualitative research has achieved an increasing role in the scientific field of EOL care [13, 14]. Due to distinctive features of the palliative care patient population (e. g. heterogeneous patient sample, low number of patients) quantitative research may not be applicable for certain research questions [13, 15]. Furthermore, the existence of heavy symptom burden as well as imminent death poses substantial ethical challenges to investigators. Qualitative methods, however, are ideal for exploring phenomena and experiences not easily captured through quantitative or objective measures, such as the nature of communication and decision-making processes [15].

Within this study particular attention was paid to competencies and prospects apt to meet the different needs and expectations of migrant cancer patients and their relatives, which were identified in a previous study [6]. The analysis focused on competencies and also on deficits and exigency as well as suggestions to improve transcultural cancer care at the EOL.

## Methods

Semi-structured interviews were conducted with staff members in oncology and PC exploring their clinical experiences with cancer patients with a migrant background and their relatives. The transcribed interviews were analyzed using thematic analysis [16] and enhanced with grounded theory techniques [17]. The study protocol was approved by the Ethics Committee of the Medical University of Vienna (963/2011).

### Sample

This analysis was part of a larger qualitative study including interviews with healthcare staff, migrant patients and their relatives focusing on the experiences with culture-sensitive PC [6]. Between July and December 2014, staff members from different departments of the Vienna General Hospital were recruited. Inclusion criteria were at least 18 years of age and experience of working with patients with a diagnosis of cancer in oncology and/or PC and migrant backgrounds. The sample was purposefully chosen to represent participants with a wide range of professional backgrounds, experiences and views. Participants from different divisions, working with patients at different illness stages were selected, i. e. oncology outpatient service, oncology day clinic, oncology inpatient ward, PC ward and radiation oncology. Additionally, participants worked in six distinct professional backgrounds, i. e. medical doctors at different hierarchy levels, nursing, spiritual care, social and honorary work and psychology. All interviewed healthcare staff members gave written and oral consent to participate in the study. Recruitment continued alongside data analysis with the goal of theoretical saturation, i. e. until additional interviews no longer generated new information and categories [17].

### Procedures

Interviews were conducted by three different researchers (SRS, TR, BS) in order to prevent interviewer bias due to individual interview styles. The topic guide started with broad and open questions about culture-sensitive care for patients with a migrant background. During the interviews, participants had a high degree of control over the conversation, allowing new topics to emerge. Follow-up prompts aimed to gain a deeper understanding about the particular influence and potential prospects of PC on the care of migrant patients and their relatives. In Austria, the majority of immigrants historically stem from the Balkans and the Middle East [2]. Therefore, if not otherwise specified, results of the interviews refer to these groups of immigrants. Interviews lasted 30–135 min. All interviews were audio recorded, transcribed verbatim, and anonymized.

### Analysis

Data analysis started with theoretical assumptions by the researchers reflecting their knowledge of the literature but without an a priori hypothesis. Interviews were coded with the software package NVivo 10 (QSR International Pty Ltd, Doncaster, Victoria, Australia). A combination of inductive and theoretically driven techniques was used to thematically analyze the data [16]. The analysis was complemented by techniques from grounded theory, including iterative inductive coding, line by line coding, constant comparison including seeking and explicitly discussing negative cases, the use of memos throughout the analytical process, as well as the use of summary tables to organize clusters of topics [17]. Themes were identified, coded, and checked for fidelity in an inductive process. This involved iterative coding, regular inspection of the data and discussion in the project team, in order to reach consensus on an increasingly refined organization of data. Later stages of analysis included a more interpretative approach, where clusters of categories partly consisted of participants’ statements and partly of interpretative labels. Alternative interpretations among the independently analyzing project group members were discussed on a regular basis resulting in the repeated adaption of an iteratively emerging framework.

## Results

Overall, 21 staff members participated in this study. The characteristics of all study participants are displayed in Table 1.

**Table 1:** Sociodemographic variables of study participants (*N* = 21)

Variable	Mean	SD
Age (years)	42 (range: 22–64)	11.41
–	***N***	**%**
*Gender*		
Female	15	(71)
*Profession*		
Physicians	8	(37)
Nurses	6	(29)
Psychologists	4	(19)
Spiritual care	1	(5)
Social worker	1	(5)
Volunteer	1	(5)
*Migrant background*		
Yes	5	(24)
*Birthplace*		
Austria: Vienna (capital) and surroundings	7	(34)
Austria: rural areas (i. e. within country migration)	8	(37)
Other countries	6	(29)

Previous results of this project [6] suggested several culture-specific differences in the clinical encounter with patients with a migrant background as compared to native patients (Table 2). The aim of this analysis was to find specific competencies and prospects of PC apt to meet these differences and fulfil the needs and wishes of migrant patients and their relatives (Table 3). Only data distinctly referring to patients with migrant background are reported. Table 4 provides example quotations to illustrate the findings. A total of four key topics of PC in the clinical encounter with migrant cancer patients and their families were found: (A) structural conditions, (B) personal conditions, (C) specific care and treatment intentions and (D) personnel requirements and attitudes.

**Table 2:** Culture-specific differences in the clinical encounter with patients with migrant background as compared to native patients

1	Social structure
2	Dealing with support
3	Language barriers and culture-specific terms
4	Models of disease
5	Expression of emotions and symptoms
6	Traditions and rituals
7	Attitude towards dying and death

**Table 3:** Specific competencies and prospects of palliative care apt to meet differences and fulfil needs and wishes of migrant patients and their relatives

	Social structure	Dealing with support	Language	Models of disease	Expression of emotions/symptoms	Traditions and rituals	Attitude toward death and dying
*A. Structural conditions of PC*							
A 1. Higher ratio of staff	–	X	–	–	–	X	–
A 2. Low number of patients per room	X	–	–	–	–	X	–
A 3. Possibility of extended hospital stays	X	X	–	X	–	–	–
A 4. Extended visiting hours	X	–	–	–	–	–	–
*B.* * P* *ersonal structures at PC wards*							
B 1. Interdisciplinary teamwork	–	X	–	–	–	–	–
B 2. Honorary workers	–	X	–	–	–	–	–
B 3. Higher status of social work and psychology	–	X	–	–	–	–	–
B 4. Wider decision-making scope for nurses	–	–	–	–	X	–	–
*C. Care and treatment intentions*							
C 1. Comprehensive treatment intention	–	–	–	X	X	–	X
C 2. Focus on needs apart from illness	–	–	–	–	–	X	–
C 3. Emphasis on end-of-life conversations	–	–	–	–	–	–	X
C 4. Providing support for relatives	X	–	–	–	–	–	–
*d. Personal requirements and attitudes*							
D 1. Close relationship to the patient	–	–	X	–	–	–	–
D 2. Knowledge about rituals in death and dying in other cultures	–	–	–	–	–	–	X
D 3. Willingness to bend rules	X	–	–	–	–	X	–
D 4. Shield patients against overwhelming relatives	X	–	–	–	–	–	–

**Table 4:** Interview topics and example quotes from participants

A. Structural conditions of PC for patients and their relatives with migrant background	
A 1. High ratio of staff	*[…] because we can be flexible and we can allow people to stick to their own rhythm instead of forcing the hospital rhythm on them. There are many people, often from more southern countries, who get up much later. Their day starts later and they go to bed later. Here (at the PC ward) that is possible, because there are more staff. Somebody will be available to do this or the other caring task any time*
A 2. Low number of patients per room	*When a patient is dying, they get a single room anyway. So they are alone in the room and it is easier to allow big groups of relatives to join and be in the room with the patient. So relatives can really take their space*
A 3. Possibility of extended hospital stays	*I* * (* *social worker) see myself as a spokesperson for those clients. Of course, they may need to go home at some point because we are not a nursing home, but there needs to be time to sort out the relevant things. So there can be a more gentle transition into home care*
A 4. Extended visiting hours	*This sometimes leads to difficulties, yes. Especially in rooms with more than one patient when the family caregivers insist on staying with the patient around the clock and then there is a lot of coming and going of people*
B. Personnel structures at PC wards	
B 1. Interdisciplinary teamwork	*I think what really helps is the interdisciplinary collaboration on the ward. It helps to really understand their situation, because we meet in the “social ward round” and everybody contributes: like how nurses perceive the situation and what it looks like for others. And often we talk about cultural differences and then e.* *g. a key nurse explains what they think the patient needs and why, and what’s up with the relatives and so on. That also helps understanding in general*
B 2. Honorary workers	*Yes, exactly, it’s like building bridges. But then they (family carers) communicate quite explicitly that there is nothing to talk about. They make very clear that they can’t deal with terminal care. And it is always more about the family, in my view especially when it comes to migrants it is more caregiver care than patient care*
B 3. Higher status of social work and psychology	*As a social worker it’s brilliant. You see, there are 12 patients and there is a structured concept for the PC ward, and it says there need to be at least 20 social work hours per week for those patients. That really opens up opportunities you don’t get on other wards like trauma surgery, orthopedics, urology or whatever. You can really understand and help patients individually*
B 4. Wider decision-making scope for nurses	*We are more autonomous. We can often act immediately where other wards need to wake up the doctor and wait for advice. We have contingency prescriptions saying what we are allowed to do or administer in this or that case. These are discussed and written down in advance and then we know what we can do, and we can decide*
C. Care and treatment intentions for patients and relatives with migrant background	
C 1. Comprehensive treatment intention	*The concept of PC in general can be difficult to convey. Often there are these people who come here not to get palliative treatment but they expect some sort of miracle cure or an assurance that everything will be mended. The message of palliative care just doesn’t hit home for them*
C 2. Focus on needs apart from illness	*On the oncology ward people are admitted for a few days to receive chemo or have some check-up, but at the PC ward goals are different. People are here for longer periods of time in order to solve various problems including social ones, and then we can of course also better tend to specific spiritual needs and necessary rituals*
	*We just deal with that more and more explicitly. After all, people are dying—that’s different to getting a piece of gut removed and going home after 3 days. Maybe some people feel irritated by large groups of visiting relatives from Turkey, but here on the palliative care ward people are dying, and that can take longer and it is a more intimate matter and it has an important effect on the family. We need to pay attention to that even if it is sometimes difficult to understand*
C 3. Emphasis on end of life conversations	*Death can be a taboo, and that may need to be respected as a cultural need. For example, there was this man from Turkey, his wife was dying and it was very difficult with the small children and everything. The man said it should not have happened that a doctor told his wife so directly that she was terminally ill. He thought that information caused her to deteriorate further, caused her to have a stroke and so on. So if there is really such a strong desire not to be told, personally I think that should be respected. But of course I know that is difficult on a palliative care ward*
C 4 Providing support for relatives	*Here at the ward that really works very well. We are all well attuned and supportive of each other, so care for relatives works on an interdisciplinary level. We’ve had lots of practice, and dealing with special needs and extraordinary demands is our daily bread, across professional boundaries*
D. Personal requirements and attitudes for working with patients and their family members with migrant background	
D 1. Close relationship to the patient	*… body language and other non-verbal signs are often very helpful. When I have a patient and I start to know him well at some point, and when I talk to him, I mean, I just need to enter the room and I can tell that he is burdened just from the way he sits on his bed. And I think, well I feel that it is possible to also bring some relief, even if they don’t speak much German, with little words, or with my own body language, that can help*
D 2. Knowledge about rituals in death and dying in other cultures	*The greatest pitfall is ignorance, not knowing about other cultures, e.* *g. about what would be a serious mistake. Some things we may take for granted in our own culture might be completely off limits in a different culture, and that is especially true when it comes to dying and dealing with a deceased person*
D 3. Willingness to bend rules	*It is difficult to define very narrow rules for PC wards. We can bend rules a bit, we are a bit different than the rest of the hospital. If it wasn’t like that our patients could be cared for anywhere. We have time and that allows us to do things that might be slightly outside regulations, or at least outside usual routines. We have discussed that countless times in team supervisions, it is a very difficult topic, and we never reached a consensus. Where are the boundaries, what should we do and what should not be tolerated? When it comes to me, I think that is an individual thing, it depends on the family, on the situation, on this relationship you have with the patient, and it can be completely spontaneous*
D 4. Shield patients against overwhelming relatives	*Well, especially with patients from Turkey that is often an issue. Families expect too much when they think the patient can tolerate 20 people in the room looking at him. That is stressful! [..] it is often necessary to set an end to scenes like that. Sometimes patients are at the verge of collapsing, especially terminally ill cancer patients, they are very tired and need lots of sleep but force themselves to stay awake for as long as visitors are there*

### A. Structural conditions of PC for patients and their relatives with migrant background

Interviewees reported several structural differences that characterize PC and distinguish it from other fields of oncology. Some of these differences were seen as particularly important to meet culture specific needs of patients with migrant background.

#### A 1. High ratio of staff

A main requirement of PC units is the high ratio of staff, i. e. fewer patients per nurse compared to other wards caring for cancer patients (e. g. oncology, surgical or radiation therapy wards). This was reported as a major advantage when dealing with migrant patients, as they were described as sometimes more demanding with higher necessary time commitment to fulfil the need for repeated conversation. Higher staff rates were also more able to recognize and accommodate culture-specific traditions and rituals.

#### A 2. Low number of patients per room

Because of the particular needs of PC, two-bed rooms and single rooms are recommended and usually available on PC units. In the majority of cases, patients in the final stage of the disease can stay in a single room together with an accompanying person. Nevertheless, migrants and their frequently large families were implicitly described as tending to be noisy and potentially disturbing to fellow patients. Hence, low patient numbers per room were perceived as protecting other patients from disturbance due to large numbers of migrant family visitors.

#### A 3. Possibility of extended hospital stays

Family members of migrant patients were reported to be particularly involved in nursing care, making it easier to discharge patients from hospital. At the same time, these patients were also reported to frequently and unnecessarily visit the outpatient service with sometimes only minor symptoms. The PC units were thought of as a place where patients could stay longer than might be medically necessary but to arrange social matters such as mobile care or instruct relatives to provide nursing care at home. This could prevent unnecessary outpatient visits and hospital readmission.

#### A 4. Extended visiting hours

Diverse social structures in migrant families were thought of as major challenge in daily clinical routine. Patients were reported to often have large families with a perceived obligation of relatives to stay overnight or take over nursing duties for the patient. The PC units do not usually have restricted visiting hours, which was regarded as especially accommodating for these circumstances. Nevertheless, some concerns existed about other patients and their need for rest.

### B. Personnel structures at PC wards

Several characteristic features were reported when thinking about the typical PC team. These characteristics included the interdisciplinary teamwork but also the expanded area of expertise and range of responsibilities of nurses.

#### B 1. Interdisciplinary teamwork

A PC team usually consists of professionals with many different backgrounds, including physicians, nurses, spiritual care, social work, psychologists and honorary workers. Close teamwork was considered especially beneficial for migrant patients, since many of these patients have trouble to navigate the health and social care system both inside and outside the hospital due to lack of information or understanding.

#### B 2. Honorary workers

The involvement of honorary workers was consistently seen as a positive feature of PC teams. As honorary workers do not have to deal with daily clinical routines, such as nursing care or the administration of treatment, they can take their time with patients and respond individually to patients’ needs and demands; however, extensive family systems of migrant patients were perceived to hinder honorary workers to directly work with patients.

#### B 3. Higher status of social work and psychology

Interdisciplinary teamwork includes the permanent availability of social workers and psychologists. Migrant patients were reported to demand such services less often but benefit strongly if received. Hence, it seemed important to repeatedly offer these crucial services. Culture-sensitive care, especially in terms of adequate psychological support overcoming language and cultural barriers was seen as insufficiently available.

#### B 4. Wider decision-making scope for nurses

Nurses repeatedly reported expanded responsibilities, including the autonomous administration of contingency medication. This was seen as a necessary level of autonomy in PC, especially when working with migrant patients, whose expression of pain and other symptoms was often more intense than that of native patients. In Austria, nurses have little autonomy for clinical decision-making and require immediate doctor’s approval for most tasks exceeding basic care. In PC institutions, however, nurses tend to enjoy a higher level of autonomy, allowing enhanced and flexible patient care as well as adding to workplace satisfaction.

### C. Care and treatment intentions for patients and relatives with migrant background

The primary objective of PC is defined as managing symptoms, including physical as well as psychosocial and spiritual needs of patients and their relatives. As the intention of treatment is no longer curative, interviewees described patients’ focus to shift to other goals and desires, in the case of migrant patients these were often traditions and rituals from their culture of origin.

#### C 1. Comprehensive treatment intention

The palliative treatment of migrant patients was consistently seen as difficult, due to different models of disease and attitudes towards EOL care. The PC entails the unconditional acceptance of subjective symptoms and their alleviation. This was seen as especially beneficial for migrant patients, who tend to express their physical and psychological symptoms more intensely than native patients. At the same time, ways to handle imminent death are culturally determined and struggles to accept a palliative instead of curative treatment intent might show up differently in migrant patients and their families. Some migrant patients and their relatives have no understanding of the concept of PC and difficulties understanding its remits and goals, which, together with language barriers, posed particular challenges to staff members.

#### C 2. Focus on needs apart from illness

Patients receiving PC were perceived to shift their attention to other goals and needs than patients in curative treatment. In migrant patients, traditions and rituals from the culture of origin were reported to gain relevance in a way that had not seemed present in earlier stages of illness. Interviewees perceived PC to have the privilege to be able to focus on these needs in addition to symptom control. Especially the encounters with families from the Balkans and the Middle East were seen as demanding in daily clinical routine. The palliative situation poses a high burden on patients’ relatives in general. This may be more visible in migrant families, which are more extended and hierarchically structured than native families and in which the expression of pain and bereavement was perceived as more intense.

#### C 3. Emphasis on EOL conversations

Interviewees reported that patients are routinely offered information about their life-threatening disease at the PC ward. Despite seen as necessary in order to provide best possible care, EOL conversations seemed challenging with migrant patients. Hindrances included potential language barriers and the lack of adequately trained translators, but most importantly insecurities about the cultural appropriateness of talking with patients about their life-limiting disease. While in Austria there is a legal requirement to inform patients in person, interviewees felt that in other cultures relatives rather than patients strived to handle this information. Interviewees placed consistent emphasis on the necessity to consider individual patients’ preferences and opinions. Relatives, interposed between staff and patients, seemed to frustrate staff efforts and restrict possibilities for adequate EOL discussions.

#### C 4 Providing support for relatives

Providing support for relatives in patient care was seen as a major task of PC. Participants felt that especially in hierarchically structured migrant families in which the head of the family was seriously ill, it was crucial to ensure the best possible care for the patients’ family. This included psychological and spiritual support, regular family meetings to inform all relatives about the current situation, and the possibility to let family members stay overnight.

### D. Personal requirements and attitudes for working with patients and their family members with migrant background

Staff members working in PC described themselves to have consciously chosen their workplace. Certain personal characteristics seemed to be necessary to provide best possible EOL care and experience job satisfaction.

#### D 1. Close relationship to the patient

Characteristics of PC units (especially the higher staff to patient ratio) but also individual willingness of staff members were reported as prerequisites to establish a close relationship with patients. This close relationship was important to understand patients at stages when they were not able to communicate anymore, e. g. through focusing on body language and meta-levels of communication. The ability to communicate with limited language possibilities was perceived as a benefit when caring for migrant patients.

#### D 2. Knowledge about rituals of death and dying in other cultures

Patients’ traditions and rituals at the EOL were perceived as dependent on cultural and religious background but at the same time highly individual. Staff members considered it crucial to know about and adequately accommodate patients’ and relatives’ needs and wishes in relation to dying. Reported ways to acquire this knowledge ranged from literature reviews and seminars, to interdisciplinary exchange in team meetings as well as simply asking the patients.

#### D 3. Willingness to bend rules

One of the frequently mentioned strategies to meet culture-specific needs of migrant patients was “bending the rules”, i. e. doing things that were slightly outside official regulations or workplace description. Examples included heating up food brought by the family, allowing more than one family member to stay overnight or performing traditions and rituals, such as lighting a candle in the moment of a patient’s death.

#### D 4. Shield patients against overwhelming relatives

Although the support of relatives was seen as important for patient care, big crowds of relatives were sometimes perceived as overwhelming and detrimental to the patients’ well-being. Some staff members considered it to be necessary to shield patients against stressful family situations, e. g. as patients might not be able to express their need for rest. Interviewees were aware that acts invading patient self-determination might be more common in the PC environment. Explicit justifications included medical reasons, e. g. patients’ fatigue or necessary treatments, or social mindfulness, e. g. other patients in the same room needing some quiet.

## Discussion

Migration as well as the cultural integration of immigrants nowadays is an issue with prominent media coverage. In the increasingly multi-ethnic societies, supporting and fostering cultural awareness is not only a political duty but also an obligation for social and healthcare systems. Importantly, cultural beliefs and needs strongly impact on QOL in cancer patients and may become crucial at the EOL [1, 3, 4, 6, 7, 11, 12, 18, 19].

In Austria, like other German-speaking countries, the majority of immigrants stem from the Balkans and the Middle East [2]. The perceived dominance of migrants with large families reflects European migration politics in the economic boom years with large numbers of guest workers, mainly from rural areas, who were joined by their families in the 1970s and 1980s. These migrants are still often found to be marginalized and fall below average in terms of sociodemographic characteristics, such as educational achievement or income [2]. Reflecting this situation, interviewees’ experiences and encounters mostly referred to these migrant populations, while solitary migrants from other countries appeared to pose less of a challenge to staff. While migrant populations differ in other societies (such as the Anglo-American countries), the structures, possibilities and prospects of transcultural PC, however, will broadly be transferable.

To our knowledge, this study provides the first insights into aspects and prospects of transcultural PC in the clinical encounter with migrant cancer patients and their families. The results may have important implications for the EOL care in this growing population. Increased attention to the importance of providing care for increasingly diverse societies, such as the Anglo-American populations in the USA, UK, Canada, Australia and New Zealand, has only been a recent development [1, 11, 12]. In German-speaking countries, such as Switzerland, Germany and Austria, a scientific focus on these processes is still evolving [2, 5, 6, 20–22].

## Clinical and social implications for end-of-life care

The results emphasize organizational features as well as individual characteristics of staff at PC wards that might benefit migrant cancer patients and their family caregivers. Importantly, the data also point towards important training needs and potentially relevant topics for reflection and good practice support in PC staff.

Structural (e. g. lower number of patients per room, extended visiting hours, lower hospital discharge pressure) as well as personal features (such as the greater ratio of staff, provision of multiprofessional teams as well as higher status of social work and psychology) create specific conditions for exploring and acknowledging possible culture-specific exigency. Through the special structural and personal conditions, gaining knowledge of individual’s beliefs and values, preferences can be achieved. Subsequently these practices can result in best possible culturally adequate care. The PC may offer a more distinctive and comprehensive treatment for migrant patients and their relatives, helping to prevent and rectify misunderstandings, stereotypes, and prejudices for patients, their families as well as healthcare staff [1, 11, 12].

In the intimate situation of imminent death, it is of special importance to reflect clinical practice for potentially more vulnerable patients such as migrants, e. g. when it comes to patient self-determination and protection. High personal commitment of individual team members, e. g. when “bending rules” for particularly needy patients and their families, might also be disruptive to team cohesion. Specific supervision and reflection might prevent misunderstandings and help PC to reach its full potential in culturally sensitive care.

According to the WHO definition, PC is an approach for the improvement of QOL not only in patients but also in their family members [10]; however, this task was perceived as particularly challenging in the work with migrant patients. In contrary to western societies, where respect for the individual autonomy is an ideal, in other populations the family or community is considered to be a unit of autonomy. Furthermore, caring for a family member is considered as an obligation as well as a matter of honour and integrity [23]. Family support and social networks are crucial factors in the psychological well-being of seriously ill and dying patients and informal caregivers play an essential role both for the patients as well as for the healthcare system [11, 12, 24]. The emphasis on care in the community rather than institutions and the preference to die at home or in the country of origin, makes informal caregivers indispensable partners of health and social care professionals [1, 11, 12].

Considering home care, these results show that migrant patients and their families often seem to have less information and access to social support and services [1, 20]. This problem should be emphasized and overcome by higher status of social work as regularly offered on PC wards. Furthermore, compared to Europe and English-speaking countries, hospices and PC services may be unavailable or unequal in resource-poor countries [1]. Subsequently, immigrants may not have had exposure to or knowledge about PC services in their home country. When confronted with PC they may have misperceptions or lowered priorities for this type of care [1]. Through culturally effective communication myths and misunderstandings about the goal of PC should be addressed early in the trajectory of the disease [1]. Importantly, speaking about the dying process itself or formal advance care planning documents may represent a severe transgression of a cultural taboo and create additional distress; however, instead of abandoning the goals of advance care planning, strategies to facilitate understanding should be sought [1].

Adequate communication was a consistently reported challenge in the work with migrant patients and their families. Hindrances such as language barriers and the lack of trained translators impede the essential need for providing culturally sensitive EOL conversations. As previously described [6, 21, 22, 25] this has been shown to be a major source of serious problems and misunderstandings in clinical consultations with patients, their relatives and healthcare providers. Some of these concerns could be minimized through efforts to boost interpreter accuracy and completeness.

The close relationship of PC professional caregivers with patients and their relatives and their personnel commitment and willingness may also harbor problems. This became especially obvious when discussing the setting of boundaries and bending of rules. Individual nurses naturally differ in their perception and enforcement of boundaries and this may lead to conflict within the team. For example, allowing large numbers of visitors to stay overnight with a dying patient may be contrary to fire department regulations for some but perceived as a natural act of kindness by others. Likewise, sending large numbers of visitors away to protect a tired patient may be both considerate and invasive.

## Limitations

An important limitation of this study is that staff members from extramural and mobile PC teams were not interviewed; however, as discussed earlier, patients and relatives with migration background often only have limited access to these special services. Racial and ethnic disparities in the utilization of healthcare services in general and in PC in particular are well-documented [4, 20, 24, 26, 27]. Some immigrants may find accessing quality care and funding for hospice, PC or other EOL services a challenging process that may be evoked by unfamiliarity with laws and regulations of the host country. Other immigrants, such as refugees or asylum seekers who have experienced violence or torture in their home countries, however, may be mistrustful of public healthcare, social services and authorities [24, 26, 27]. Clarifying potential barriers to accessing EOL services in the Austrian healthcare system will have to be an important aim of future studies.

## Conclusion

Due to the trend of increasingly multi-ethnic societies, healthcare professionals have to face the challenges in delivering evidence-based culturally competent care. As demonstrated by these data, PC is an important and invaluable approach in the clinical encounter with migrant cancer patients and their relatives due to its structural, personal and personnel features; however, identification of health inequalities in the delivery of PC as well as monitoring and ensuring cultural competency will have to be a critical mandate in the future.
